# Torticollis as Presentation for Atypical Kawasaki Disease Complicated by Giant Coronary Artery Aneurysms

**DOI:** 10.1155/2018/4236264

**Published:** 2018-10-08

**Authors:** Tracey Dyer, Paul Dancey, John Martin, Suryakant Shah

**Affiliations:** Department of Pediatrics, Memorial University, 300 Prince Phillip Drive, St. John's, NL, Canada A1B 3V6

## Abstract

Kawasaki disease (KD) is an acute systemic vasculitis of childhood. The diagnosis can be made in a patient who presents with a prolonged high fever and meeting at least four of five criteria including polymorphous rash, mucosal changes, extremity changes (including swelling and/or palmar and plantar erythema), bilateral nonsuppurative conjunctivitis, and unilateral cervical lymphadenopathy. Atypical KD refers to patients who have not met the full criteria and in whom atypical features may be present. We discuss a case of a 6-year-old male who presented to the Emergency Department with torticollis. A series of investigations for elevated inflammatory markers revealed dilated coronary artery aneurysms on echocardiogram, and thus he was diagnosed with atypical KD. His only other criteria were bilateral nonsuppurative conjunctivitis and a prior brief febrile illness. He was treated with high-dose intravenous immune globulin (IVIG) and low-dose aspirin. Low-molecular-weight heparin and atenolol were added due to the presence of giant aneurysms.

## 1. Introduction

Kawasaki disease (KD) is an acute systemic vasculitis of childhood diagnosed by high fever for 5 days accompanied by at least four of the following symptoms: polymorphous rash, mucosal changes (including dry, cracked lips and strawberry tongue), extremity changes (including palmar and/or plantar erythema, swelling, and desquamation), bilateral nonsuppurative conjunctivitis, and cervical lymphadenopathy [1]. It is the second most common vasculitis of childhood with a peak age of 2-3 years and rarely seen above the age of 7 years. KD is now the most common cause of acquired heart disease in children in developed countries [2]. Atypical KD refers to patients who have not met the full criteria and in whom atypical features may be present [3]. Risk factors for development of coronary artery aneurysms with KD include prolonged fever, prolonged elevation of inflammatory markers, age younger than 1 year or older than 6 years at onset, and male gender [1]. KD is treated with aspirin and intravenous immune globulin (IVIG) which reduces the incidence of coronary artery involvement from approximately 25% to less than 4% [4].

## 2. Case Presentation

A previously healthy 6-year-old boy presented to a pediatric hospital with a 3-week history of torticollis. He had symptoms of an upper respiratory tract infection four weeks prior and had 2 days of documented fever at home during that time. He had been treated with a 7-day course of amoxicillin by the primary care physician for suspected streptococcal pharyngitis. Four days into the course of antibiotics, he woke up from sleep with pain on the left side of his neck. Despite taking ibuprofen and acetaminophen, he presented to the Emergency Department 3 weeks later due to persisting torticollis. Pain was worse with movement. There was no history of head/neck trauma. At the time of presentation, the infectious symptoms had resolved. Some fatigue was noted but he remained generally active, continuing to play hockey. There was no history of rash, peripheral joint pain, or weight loss. Past medical history and family history were unremarkable.

On examination, the patient was afebrile with normal blood pressure for age and a maximum heart rate of 110 beats per minute. The patient's head was tilted to the right with chin rotation to the left. No lymphadenopathy or masses were noted on palpation of the neck. There was no tenderness to palpation of bilateral sternocleidomastoid muscles. There was a limited range of motion in all planes of rotation of the neck secondary to pain, particularly in lateral flexion. Bilateral injected conjunctivas were present. The oropharynx was normal with no erythema or mucus membrane changes. Cardiovascular exam revealed normal peripheral pulses, a quiet precordium with normal heart sounds, and no murmur. Respiratory exam was normal. The abdomen was soft with no distension, tenderness, or hepatosplenomegaly. There were no bruits heard on auscultation of major vessel regions. There were no rashes or desquamation of the skin. Neurological exam was normal.

At the time of presentation, laboratory investigations revealed an elevated white blood cell count of 17.4 × 10^9^/L with a neutrophil count of 14.1 × 10^9^/L. Hemoglobin was normal for age at 110 g/L. Inflammatory markers were elevated including platelet count of 860 × 10^9^/L and CRP of 38.5 mg/L. Renal function (BUN and creatinine) and liver function (ALP and ALT) were normal for age. Because of the unexplained elevated white blood cell count and evidence of inflammation, a chest X-ray was performed which revealed normal lung fields but an enlarged cardiac silhouette. X-ray of the cervical spine was normal with no atlantoaxial rotary subluxation demonstrated. Ultrasound of the neck revealed mild thickening of the left sternocleidomastoid muscle and no lymphadenopathy. Abdominal ultrasound with Doppler was normal.

**Figure 1:**
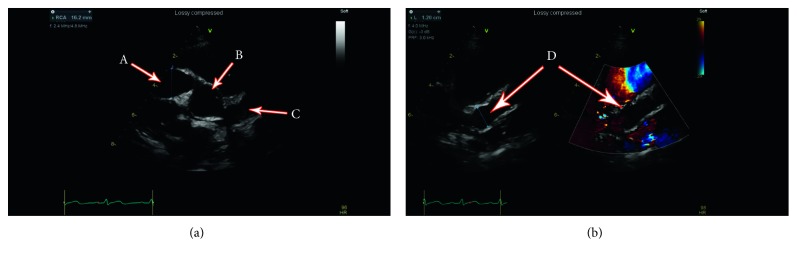
Echocardiogram: (a) Massive ectasia and aneurysmal dilatation of the right coronary artery (16 mm × 17 mm) (A), aorta (B), and left coronary artery (C). (b) Massive ectasia and aneurysmal dilatation of left main artery (13 mm × 13 mm) (D).

Additional investigations included a normal throat swab for group A streptococci and a negative anti-streptolysin O antibody titer. High-sensitivity troponin was elevated to 176 ng/L. Creatinine kinase was normal. ANCA was normal. Electrocardiogram showed normal sinus rhythms without evidence of chamber hypertrophy. The patient underwent an echocardiogram to further characterize the enlarged cardiac silhouette identified on the chest X-ray. This revealed massive ectasia and aneurysmal dilatation of the right coronary artery, left main artery, left anterior descending artery, and circumflex arteries, as seen in Figure 1. Left ventricular function was normal. The aortic arch was normal as were the proximal neck vessels.

Because of the dilated coronary aneurysms, the patient was diagnosed with KD. Despite lack of fever, given the evidence of ongoing inflammation and initial presence of bilateral nonsuppurative conjunctivitis, in addition to the coronary artery changes, the patient was treated with high-dose IVIG (2 g/kg) and started on daily low-dose aspirin. Low-molecular-weight heparin was started as antithrombotic therapy and once stabilized, daily atenolol was initiated. Activity was restricted as much as possible.

Inflammatory markers were followed. Platelets revealed a peak of 952 × 10^9^/L and CRP a peak of 54.6 mg/L. After treatment, both platelet and CRP levels normalized.

**Figure 2:**
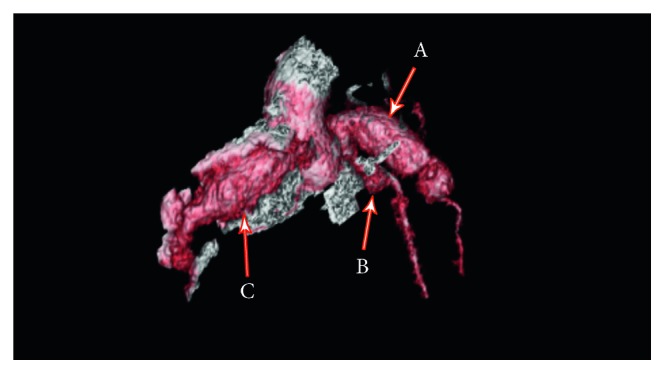
CT angiogram: there is a long fusiform aneurysm involving the proximal LAD measuring approximately 12 mm × 12 mm and extending over a length of 2.3 cm (A). The left circumflex is aneurysmal proximally measuring approximately 5 mm × 5.5 mm (B). There is a long fusiform aneurysm of right coronary artery measuring 16 mm × 16 mm and extending over a length of at least 3.2 cm (C).

The patient's neck pain and the limited range of movement resolved immediately after treatment, as did the bilateral conjunctivitis. The patient was stable and appeared well at time of discharge. His aspirin, low-molecular-weight heparin, and atenolol were continued. The CT angiogram performed after discharge revealed massively dilated and aneurysmal coronary arteries, as shown in Figure 2.

In follow-up cardiology and rheumatology clinics, he has been doing well with no further neck pain or stiffness. He did not develop desquamation during follow-up, and the repeat echocardiogram one month after discharge was unchanged. He will continue long-term anticoagulation therapy with low-dose heparin with a target level greater than 0.5 IU/ml. He will also continue low dose aspirin and atenolol. His family was advised to have the annual influenza vaccine.

## 3. Discussion

Our patient was diagnosed with KD after dilated coronary artery aneurysms were found on the echocardiogram. He had a history of fever for two days that occurred three weeks prior to presentation, and no further fevers were documented or recognized by his parents. The only criteria of KD met on history and examination at presentation was bilateral nonsuppurative conjunctivitis. Blood work did reveal evidence of ongoing inflammation. Risk factors for KD with coronary involvement were male gender and a delayed presentation prior to diagnosis. It is possible that he had fever longer than the reported two days as the parents had not measured it regularly at home. Presumably, the inflammatory markers had been elevated for up to three weeks prior to diagnosis; however, no blood work had been performed during the initial febrile period.

Although unusual, there have been several reports in the literature of KD presenting as torticollis or neck tilt. Different pathophysiologies have been described including KD associated with Grisel's syndrome, a rare, nontraumatic atlantoaxial subluxation [5]; KD with retropharyngeal edema and arthritis of the small joints in the head and neck region [6]; and KD with severe cervical spine and bilateral temporomandibular joint arthritis [7]. Our patient did not have any other signs of arthritis, and there was no cervical lymphadenopathy to explain the torticollis. During the admission, an X-ray of the cervical spine and ultrasound of the neck did not reveal any underlying pathology. The torticollis resolved after treatment with IVIG and aspirin and did not recur.

Despite giant coronary aneurysms, our patient has remained well since discharge from hospital and is closely followed by Cardiology and Rheumatology. He has had no further neck pain or stiffness and has not developed any further symptoms including desquamation or arthritis. Repeat echocardiograms have remained stable. He will require long-term anticoagulation therapy.

Our patient was brought to medical attention due to his torticollis. While the exact reason for the torticollis is unclear, we feel it is important to raise the awareness of this rare manifestation of Kawasaki disease.
